# α2-Adrenergic Receptor in Liver Fibrosis: Implications for the Adrenoblocker Mesedin

**DOI:** 10.3390/cells9020456

**Published:** 2020-02-18

**Authors:** Ute A. Schwinghammer, Magda M. Melkonyan, Lilit Hunanyan, Roman Tremmel, Ralf Weiskirchen, Erawan Borkham-Kamphorst, Elke Schaeffeler, Torgom Seferyan, Wolfgang Mikulits, Konstantin Yenkoyan, Matthias Schwab, Lusine Danielyan

**Affiliations:** 1 Department of Clinical Pharmacology, University Hospital of Tuebingen, 72076 Tuebingen, Germany; schwinghammer.ute@gmail.com (U.A.S.); Matthias.Schwab@ikp-stuttgart.de (M.S.); 2 Department of Medical Chemistry, Yerevan State Medical University, 0025 Yerevan, Armenia; magda.melkonyan@meduni.am (M.M.M.); h-lilit@live.com (L.H.); 3 Dr. Margarete Fischer-Bosch-Institute of Clinical Pharmacology, 70376 Stuttgart, Germany, and University of Tuebingen, 72074 Tuebingen, Germany; Roman.Tremmel@ikp-stuttgart.de (R.T.); Elke.Schaeffeler@ikp-stuttgart.de (E.S.); 4 Institute of Molecular Pathobiochemistry, Experimental Gene Therapy and Clinical Chemistry, RWTH University Hospital Aachen, 52074 Aachen, Germany; rweiskirchen@ukaachen.de (R.W.); eborkham@ukaachen.de (E.B.-K.); 5 H. Buniatian Institute of Biochemistry, National Academy of Sciences of the Republic of Armenia (NAS RA), 0014 Yerevan, Armenia; seferyant@yahoo.com; 6 Department of Medicine I, Institute of Cancer Research, Comprehensive Cancer Center, Medical University of Vienna, 1090 Vienna, Austria; wolfgang.mikulits@meduniwien.ac.at; 7 Department of Biochemistry and Neuroscience Laboratory, Yerevan State Medical University, 0025 Yerevan, Armenia; konstantin.yenkoyan@meduni.am; 8 Department of Biochemistry and Pharmacy, University of Tuebingen, 72076 Tuebingen, Germany

**Keywords:** α2-adrenoceptors, norepinephrine, mesedin, hepatic stellate cells, sinusoidal endothelial cells, liver fibrosis, sinusoidal permeability

## Abstract

The noradrenergic system is proposed to play a prominent role in the pathogenesis of liver fibrosis. While α1- and β-adrenergic receptors (ARs) are suggested to be involved in a multitude of profibrogenic actions, little is known about α2-AR-mediated effects and their expression pattern during liver fibrosis and cirrhosis. We explored the expression of α2-AR in two models of experimental liver fibrosis. We further evaluated the capacity of the α2-AR blocker mesedin to deactivate hepatic stellate cells (HSCs) and to increase the permeability of human liver sinusoidal endothelial cells (hLSECs). The mRNA of α2a-, α2b-, and α2c-AR subtypes was uniformly upregulated in carbon tetrachloride-treated mice vs the controls, while in bile duct-ligated mice, only α2b-AR increased in response to liver injury. In murine HSCs, mesedin led to a decrease in α-smooth muscle actin, transforming growth factor-β and α2a-AR expression, which was indicated by RT-qPCR, immunocytochemistry, and Western blot analyses. In a hLSEC line, an increased expression of endothelial nitric oxide synthase was detected along with downregulated transforming growth factor-β. In conclusion, we suggest that the α2-AR blockade alleviates the activation of HSCs and may increase the permeability of liver sinusoids during liver injury.

## 1. Introduction

Chronic liver disease leads to the dysregulation of various neuroendocrine systems, including the adrenergic system. A significant systemic increase in norepinephrine (NE) and epinephrine in patients with liver cirrhosis and portal hypertension has been known since the 1980s (for a review, see Reference [1]). Moreover, NE was reported to exert strong profibrogenic effects in the liver and in hepatic stellate cells (HSCs) in vitro [2] and in vivo [3]. Despite this evidence of the strong involvement of the noradrenergic system in the pathogenesis of liver fibrosis, the functional role of the α-adrenergic receptors (α-ARs) remains largely unexplored. The vasoactive effects of adrenergic receptors on portal hypertension can consequently accelerate the progression of hepatic fibrosis. Moreover, the fact that the majority of parenchymal and nonparenchymal cells in the liver do express the main subclasses of adrenergic receptors, including α2-AR [4,5,6,7], hint at the potential direct effects of this system at the cellular level. Among the adrenergic receptors, the α2 adrenergic receptor (α2-AR) subclass has been rarely investigated in the liver. Idazoxan, the main classic α2-adrenoblocker, has been shown to exert protective antifibrotic features in experimental liver fibrosis [8]. Thus, it is of potential interest to elucidate the mechanisms behind the antifibrogenic effects of the α2-AR blockade in general and to explore the potential therapeutic value of the novel α2-adrenoblocker mesedin in liver fibrosis/cirrhosis in particular. Mesedin has been previously shown to possess neuroprotective features in vivo and in vitro. In a model of focal ischemia, mesedin improved memory and anxiety symptoms and decreased oxidative stress markers in brain tissue [9,10]. In astroglial primary culture of normal and Alzheimer’s mouse model (3xTg-AD) brains, the increased survival of neurons, upregulated neurogenesis markers, and anti-inflammatory cytokines have been observed upon treatment with mesedin [11].

The role of α-ARs in healthy and diseased livers has been controversial in the literature. The sympathetic nervous system has been suggested to control oxidative stress during carbon tetrachloride (CCl_4_) induced liver injury through α-adrenergic signaling. This is evidenced by the protective effects of a nonselective α1 and α2 blocker (phentolamine) in CCl_4_-treated mice, which were reflected by decreased tissue necrosis, lactate dehydrogenase (LDH), liver enzymes, and hepatic lipid peroxidation [12].

Several profibrogenic factors are controlled by NE in liver cells. NE stimulates tumor necrosis factor-α (TNFα) secretion from Kupffer cells, which is inhibited by the α2-AR inhibitor yohimbine [4,7]. NE induces human hepatic stellate cell (HSC) proliferation and increases the expression of collagen-1α2 via transforming growth factor -β (TGFβ) [13]. On the other hand, both deleterious and protective effects from the α2-AR blockade have been reported in experimental models of liver injury. Xuanfei and colleagues demonstrated protection against the progression of hepatic fibrosis by the α2-AR blocker idazoxan [8]. In contrast, a recent in vivo study by Sha and colleagues showed that the antiapoptotic and antioxidative effects of the α2-AR agonist dexmedetomidine in the livers of rats with lipopolysaccharide-induced oxidative stress are reversed by the α2-AR antagonist atipamezole [14].

In light of the aforementioned controversial discussion around the role of α2-ARs in liver fibrosis/cirrhosis, we sought to investigate the influence of the novel α2-AR blocker mesedin (2-(2-methyl-amino-thiozolyl)-1,4-benzodioxane hydrochloride) [15] on the two main nonparenchymal liver cell types, hepatic stellate cells and liver sinusoidal endothelial cells, which are key players in the fibrotic reorganization of liver tissue and the permeability of the blood-tissue barrier during cirrhosis. Mesedin was used as a novel α2-adrenoblocker because of its selectivity to α2-adrenoreceptors and its lower toxicity compared to known structural analogs, such as idazoxan (as previously discussed in Reference [11]). Notably, the levels of α1-, β1-, and β2-AR have been previously assessed in HSCs in vitro [16], while the influence of liver injury and HSC activation on α2-AR expression remains largely unexplored. Therefore, the levels of α2-AR were analyzed in the livers of mice with CCl_4_- or bile duct ligation (BDL)-induced fibrosis and in culture-activated HSCs with and without the α2 blocker mesedin.

## 2. Materials and Methods

### 2.1. Cell Cultures

Murine HSCs (M1-4HSCs) and a human hepatic sinusoidal endothelial cell (hLSEC) line were grown in Dulbecco’s Modified Eagle’s Medium (DMEM) with high glucose (4.5 g/L) containing either 5% (for hLSECs) or 10% fetal calf serum (for M1-4HSCs), 1% nonessential amino acids (only for M1-HSCs), 100 U/mL penicillin, and 100 µg/mL streptomycin (Gibco, Thermo Fisher Scientific, Darmstadt, Germany). Cells were kept at 37 °C in an atmosphere containing 5% CO_2_.

For Western blot analyses, M1-4HSCs and hLSECs were seeded into 150-cm^2^ culture flasks at a density of 2 × 10^6^ cells/flask. To culture the hLSECs, flasks precoated with collagen 1 (ME04043, Corning, Kennebunk, ME, USA) were used. After 24 h, adherent cells were incubated for 48 h with or without 10 µM mesedin (dissolved in phosphate-buffered saline). Mesedin has been previously characterized as a selective α2-AR blocker [15].

For immunofluorescence analyses, M1-4HSCs and hLSECs were placed on coverslips in a petri dish at a density of 100,000 cells/coverslip. For hLSECs, the cover slips were precoated with collagen 1. After 24 h, adherent cells were incubated for 48 h with or without 10 µM mesedin.

### 2.2. Animal Models

All animal experiments were approved by the Local Institutional Committee for Animal Welfare in Aachen (LANUV, approval number: Az. 84-02.04.2012.A092, Recklinghausen, Germany). For chronic liver injury tests, we employed 6–8-week-old female C57BL/6J mice (Charles River, Sulzfeld, Germany), which were subjected to an intraperitoneal injection of 0.8 µL/g body weight CCl_4_ in mineral oil or vehicle twice weekly for 4 weeks, as described previously [17]. The mice were then euthanized and sacrificed under isoflurane, and blood samples were taken for standard liver function tests. Liver specimens were snap-frozen in liquid nitrogen and kept at −80 °C for protein and RNA isolation.

For BDL, eight-week-old male C57BL/6J mice were used. The animals were separated into BDL and sham-operated (SO) groups (*n* = 6 each). A common BDL procedure was performed following standardized protocols [18]. After 4 weeks, the mice were sacrificed, and the livers were snap-frozen in liquid nitrogen and stored at −80 °C.

### 2.3. RNA Isolation and Quantification

High-quality RNA from mouse liver tissue or mouse M1-4HSCs was extracted using a mirVana miRNA Isolation Kit (Thermo Fisher Scientific, Waltham, MA, USA). Per sample, 1 µg of total RNA was transcribed to cDNA using a high-capacity cDNA reverse transcription kit with an RNase inhibitor (according to the manufacturer’s instructions) (Applied Biosystems, Foster City, CA, USA). cDNA measurements of α-smooth muscle actin (α-SMA) and the α2-AR subtypes α2a-, α2b-, and α2c-AR (encoded by *Adra2a*, *Adra2b*, and *Adra2c* as well as *Gapdh* (Glycerinaldehyd-3-phosphat-Dehydrogenase) as a housekeeping gene) were performed using a 7900 Real-Time PCR System (Applied Biosystems) and predesigned TaqMan gene expression assays (see Table 1; Thermo Fisher Scientific). The ΔΔCT-method (relative expressiondifference between the cycle threshold of treatment vs control) [19] was applied to calculate the relative quantity (RQ) of target gene mRNA normalized to GAPDH.

### 2.4. Western Blot Analysis

For homogenization of the liver tissue and harvested cells, ice cold lysis buffer (300 mM NaCl, 50 mM Tris, 2 mM MgCl_2_, 0.5% NP40) containing “complete protease inhibitor” (Roche, Mannheim, Germany) was used. A DC Protein assay (Bio-Rad, Hercules, CA, USA) was employed to determine the total protein amount. Proteins were fractionated by SDS/PAGE (12% acrylamide) and transferred onto PVDF (Polyvinylidenfluorid) membranes (EMD Millipore, Billerica, CA, USA). Membranes were then blocked in 5% BSA (bovine serum albumin) (Albumin Fraction V, protease-free, Roth, Germany) in TBST (Tris-buffered saline with Tween20) for 1 h and incubated overnight at 4 °C with primary antibodies diluted in 5% BSA (see Table 2). Membranes were incubated with Cy3/Cy5-conjugated antibodies for 3 h at room temperature (RT) to visualize the antibody binding. Protein bands were detected by fluorescence detection systems (Bio-Rad, Hercules, CA, USA). Densitometric analyses and imaging were performed with a VersaDocTM 4000 MP imaging system (Bio-Rad). Data were normalized to the respective densitometric values of the loading controls (GAPDH).

### 2.5. Immunofluorescence Analyses

For immunofluorescence analyses, hLSECs and M1-4HSCs were each grown on coverslips and incubated with or without mesedin, as described in Section 2.1. The cells were then fixed in 4% Paraformaldehyde (PFA) for 15 min, washed 3 times with PBS, and incubated for 1 h at RT (Table 2). Antibodies were diluted in PBS. After incubation with primary antibodies, cells were washed 3 times with PBS, incubated with a corresponding fluorochrome-linked secondary antibody in the dark for 1 h at RT, and then washed twice with PBS and once with PBS containing 0.1% Triton^®^ X-100 (Sigma, Taufkirchen, Germany). The cells were then covered with Vectashield mounting medium (Vector Laboratories Burlingame, Burlingame, CA, USA) containing 4′,6 diamidino-2-phenylindole (DAPI) and were dried and stored at −20 °C. As for negative controls, samples were treated with secondary antibodies only.

Immunofluorescence staining was evaluated by fluorescence microscopy using an Olympus BX51 Microscope (Olympus Optical Co. Europe, Hamburg, Germany). Images were acquired using the digital camera F-View II and processed by the software Analysis DOKU^®^ (Soft Imaging System GmbH, Leinfelden-Echterdingen, Germany).

Primary and secondary antibodies were applied in concentrations in accordance with the manufacturer’s information.

### 2.6. Statistical Analyses

Data were analyzed using two-tailed Student’s *t*-test with GraphPad Prism Software (GraphPad Software Inc, La Jolla, CA, USA). A threshold of *p* < 0.05 was considered significant.

## 3. Results

### 3.1. α2 Receptors are Upregulated in Fibrotic/Cirrhotic Livers

**Figure 1:**
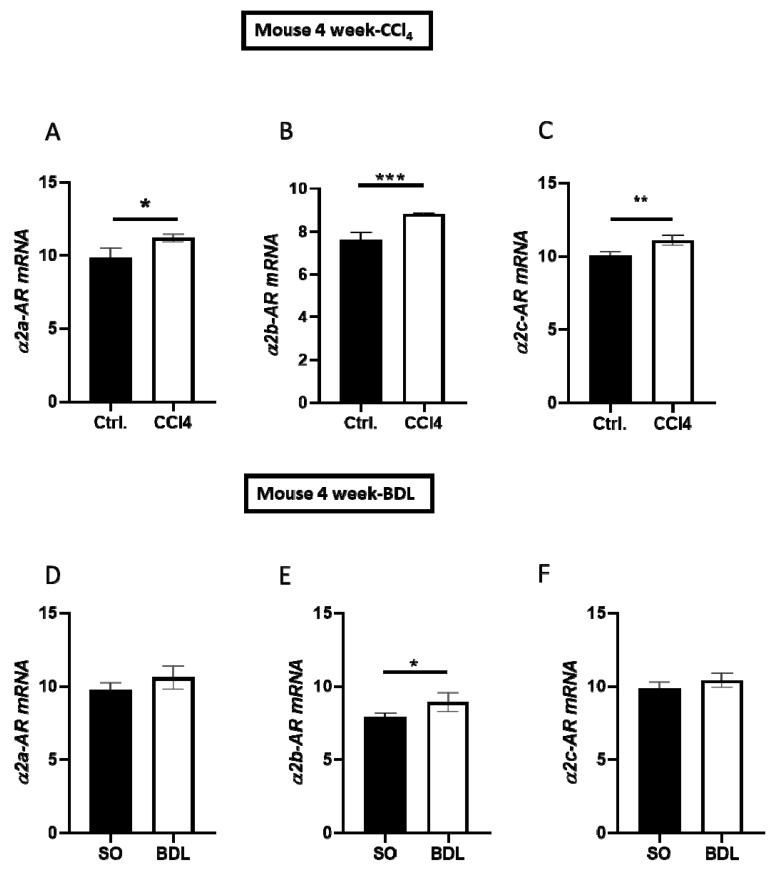
Expression of α2 adrenergic receptor (α2-AR) subtypes in carbon tetrachloride (CCl_4_)-treated and bile duct-ligated (BDL) mouse models of liver fibrosis/cirrhosis. (**A**–**C**) Expression of α2a-AR, α2b-AR, and α2c-AR mRNA in liver tissues from mice treated with CCl_4_ and controls, measured after 4 weeks using RT-qPCR. (**D**–**F**) Hepatic expression of α2a-AR, α2b-AR, and α2c-AR in mice after 4 weeks of BDL vs a sham operation (SO), measured using RT-qPCR. mRNA levels are shown as the mean ± standard error of the mean (SEM) (*n* = 4). Differences between both groups were analyzed using Student’s *t*-test. Statistical significance is indicated as follows: *p* < 0.05 (*); *p* < 0.01 (**); *p* < 0.001 (***).

To study the impact of fibrotic/cirrhotic injury on hepatic α2-AR expression, we analyzed three main subtypes of α2-ARs (α2a, α2b, and α2c) using qPCR in the livers of mice four weeks after BDL or CCl_4_ treatment vs the respective controls (Figure 1A–F). While all three receptor subtypes were uniformly upregulated after CCl_4_-induced fibrosis (cf. ctrl. vs CCl_4_ in Figure 1A–C), only α2b-AR was significantly higher than the control group in terms of BDL (Figure 1E).

### 3.2. Mesedin Decreased the Expression of α1, α2a, and α2b Receptors in HSCs

**Figure 2:**
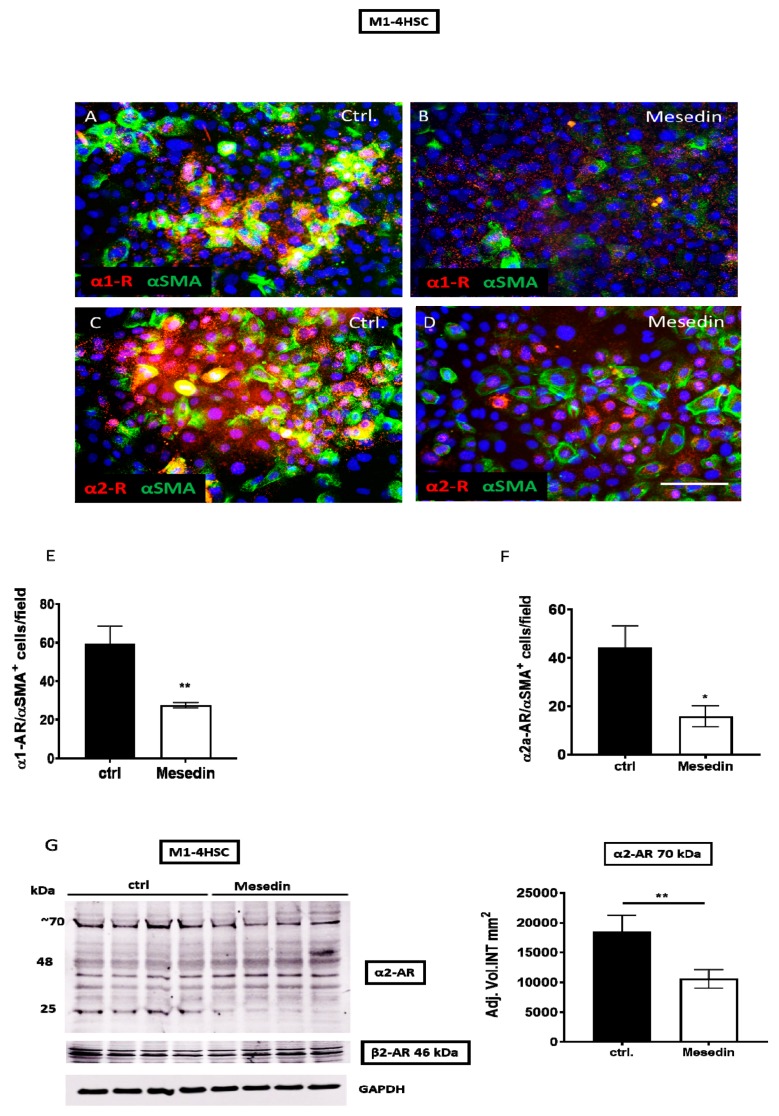
Influence of mesedin on the expression of α-adrenergic receptors in murine hepatic stellate cells (M1-4HSCs). (**A**–**D**) Immunofluorescence analysis of α1/α-ARs (red) and α-SMA (green) in M1-4HSCs treated with 10 µM mesedin vs untreated controls (ctrl.). The scale bar corresponds to 100 µm, and the cell nuclei were stained with DAPI. (**E**,**F**) Quantification of α1-AR^+^/α-SMA^+^ and α2-AR/α-SMA^+^ M1-4HSCs upon incubation with mesedin vs the control (ctrl). (**G**) Western blot and densitometric analysis of α2-AR and β2-AR in M1-4HSCs with and without mesedin. Student’s *t*-test, *n* = 4, *p* < 0.05 (*), *p* < 0.01 (**).

Next, we assessed the influence of mesedin, mediated by α2 blockade, on the expression of α2 receptors in HSCs in vitro. We used M1-4HSC cells, which displayed key features of the intermediate activation of HSCs, which were reflected by α-smooth muscle actin (α-SMA), pro-collagen I expression, and the capacity to undergo a TGF-β-induced transition into a myofibroblastic cell type [20]. In M1-4HSCs, a tendency for a decreased number of α1-positive cells was observed in mesedin-treated cultures (Figure 2A,B). A quantification of α1-positive cells determined that this difference was statistically significant (Figure 2E). The expression of α2-AR was also significantly downregulated by mesedin, which was reflected by reduced intensity in the staining (Figure 2C,D) and by the number of α2-positive M1-4HSCs (Figure 2F). A densitometric analysis of the α2a-AR Western blot (Figure 2G) revealed a decrease in the ~70-kDa band, which most likely represented a homodimer or glycosylated α2a-AR [21], whereas the β2-AR expression in mesedin-treated cells was equal to that of the control.

**Figure 3:**
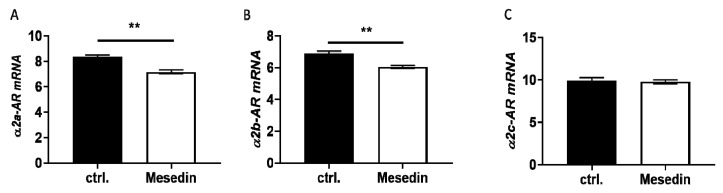
Altered expression of α2-AR in M1-4HSCs due to mesedin-treatment. (**A**–**C**) Quantification of three major subtypes of α2-AR (α2a-AR, α2b-AR, and α2c-AR) under mesedin treatment in comparison to control cultures (ctrl.) using RT-qPCR. Student’s *t*-test, *n* = 6, *p* < 0.01 (**).

At the mRNA level, only the α2a and α2b receptors were significantly decreased in mesedin-treated M1-4HSCs (Figure 3A,B), while α2c-AR remained unchanged (Figure 3C).

### 3.3. Antifibrotic Effects of Mesedin

**Figure 4:**
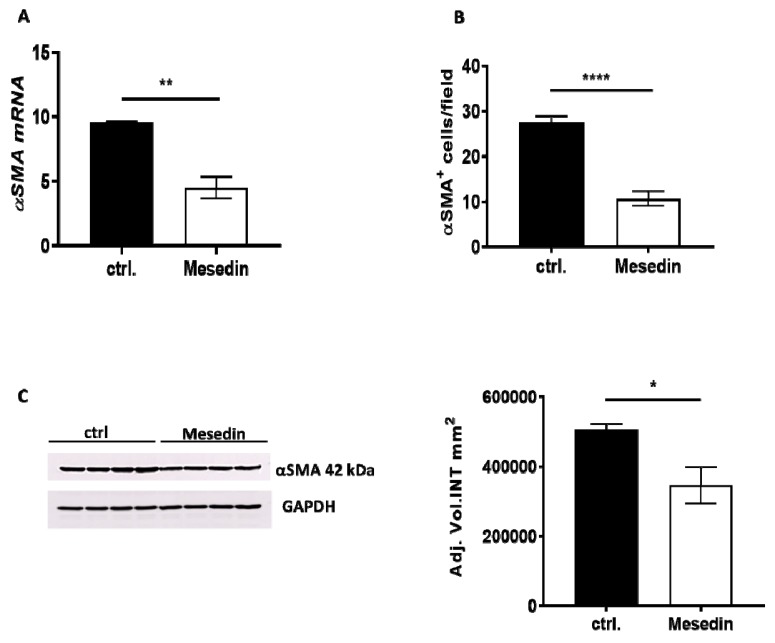
Influence of mesedin on α-smooth muscle actin (α-SMA) expression in M1-4HSCs. **(A)** Quantification of α-SMA mRNA in mesedin-treated M1-4HSCs vs controls (ctrl.), *n* = 6. (**B**) Quantification of α-SMA^+^ M1-4HSCs with and without mesedin, *n* = 5. (**C**) Western blot and densitometric analysis of α-SMA in M1-4HSCs treated with mesedin, *n* = 4. Student’s *t*-test; data are shown as the mean ± SEM, *p* < 0.05 (*), *p* < 0.01 (**), *p* < 0.0001 (****).

The decrease in α-receptors was concomitant with the downregulation of α-SMA mRNA (Figure 4A) and a reduced number of α-SMA-positive M1-4HSCs treated with mesedin (Figure 4B). These data were also confirmed by reduced α-SMA protein expression in the Western blot analysis (Figure 4C).

**Figure 5:**
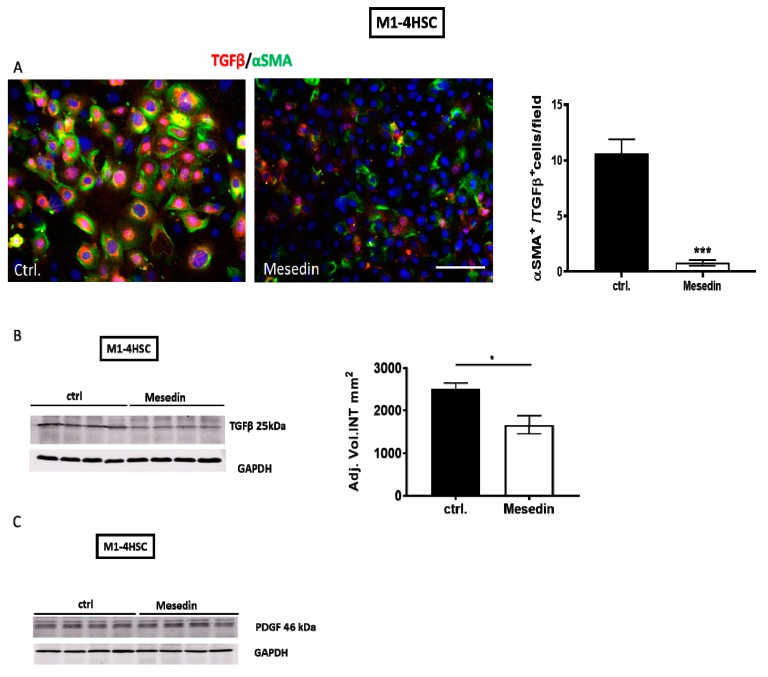
Transforming growth factor-β (TGF-β) and platelet-derived growth factor (PDGF) expression in mesedin-treated M1-4HSCs. (**A**) TGF-β (red) and α-SMA (green) staining of M1-4HSCs under control conditions (ctrl.) and incubation with mesedin. Cell nuclei were stained with DAPI; scale bar 100 µm. Quantification of α-SMA/TGF-β-positive M1-4HSCs (*n* = 9, *p* < 0.001 (***), Student’s *t*-test; data shown as the mean ± SEM). (**B**) TGF-β expression as analyzed by Western blot and a corresponding densitometric analysis of M1-4HSCs treated with mesedin vs the control (ctrl.) (*n* = 4, *p* < 0.05 (*), Student’s *t*-test, data shown as the mean ± SEM). (**C**) PDGF Western blot of M1-4HSCs with and without mesedin (ctrl.), *n* = 4.

Immunofluorescence and Western blot analyses revealed a decrease in TGF-β/α-SMA-positive HSCs (cf. ctrl. vs mesedin in Figure 5A) and in the expression level of the protein in mesedin-treated HSCs (Figure 5B). PDGF was equally expressed in mesedin-treated cells compared to the control (Figure 5C).

### 3.4. Influence of the α2 Blockade on Permeability Marker Expression in hLSECs

**Figure 6:**
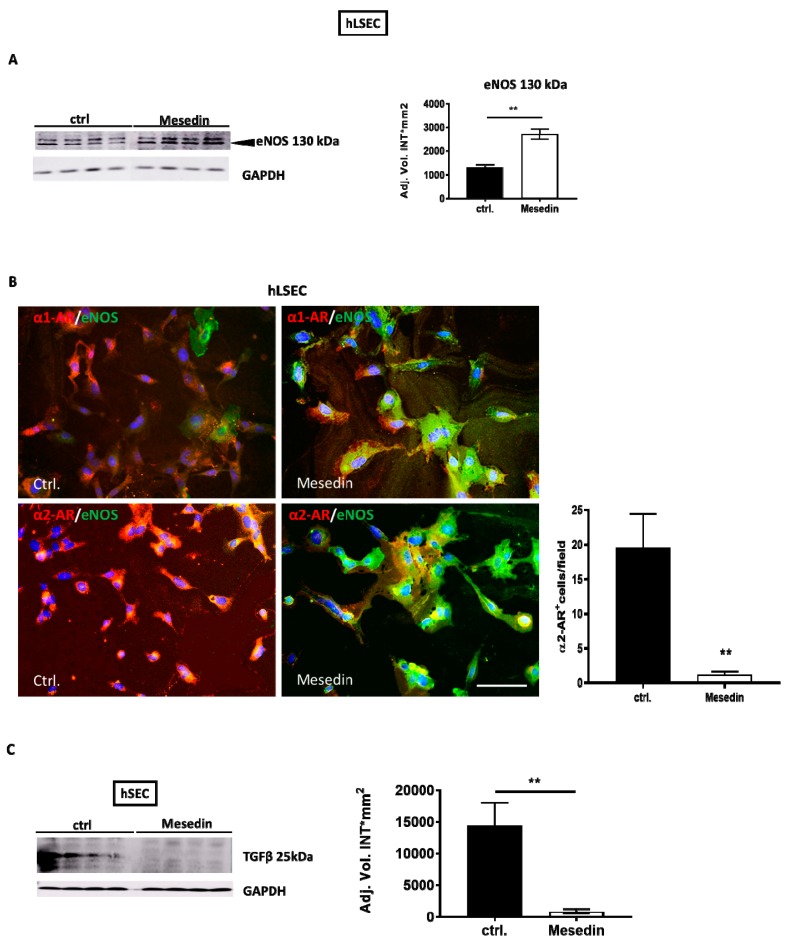
Impact of mesedin on the permeability of human liver sinusoidal endothelial cells (hLSECs). (**A**) Western blot and densitometric analysis of eNOS in hLSECs with and without (ctrl.) mesedin, *n* = 4. (**B**) Immunofluorescence analysis of α1-AR vs α2-AR (red) and eNOS (green) in hLSECs. Cell nuclei were stained with DAPI. Scale bar corresponds to 100 µm. Quantification of α2-AR-positive cells in hLSECs (*n* = 6, *p* < 0.01 (**), Student’s *t*-test, data shown as the mean ± SEM). (**C**) Western blot and densitometric analysis of TGF-β in hLSECs 48 h after incubation with and without (ctrl.) mesedin. Student’s *t*-test, *p* < 0.01 (**).

Acknowledging the importance of liver sinusoidal endothelial cell fenestration in the maintenance of normal liver function and its decrease during fibrosis/cirrhosis [22], we investigated the role of α2-ARs in the permeability and differentiation of hLSECs [23]. Here, hLSECs incubated with mesedin enhanced the expression of eNOS (Figure 6A,B). Interestingly, the high level of eNOS coincided with the decreased intensity of α1- and α2-receptors in hLSECs (Figure 6B). Finally, given that TGF-β produced by hLSECs may contribute to the activation of HSCs and may negatively influence the differentiation of hLSECs, we analyzed the impact of mesedin on TGF-β expression in hLSECs. Western blot analyses demonstrated decreased active TGF-β (25kDa) in mesedin-treated hLSECs (Figure 6C).

## 4. Discussion

The present study reports for the first time the antifibrotic effects of the novel α2-AR blocker, mesedin, in HSCs and its capacity to increase the permeability marker eNOS in hLSECs. In chronic CCl_4_-induced fibrosis, all subtypes of α2-AR (α2a, α2b, and α2c) were increased. In contrast, in acute BDL-caused injury, only α2b was elevated. HSCs responded to mesedin with a decrease in key fibrotic markers such as TGF-β and α-SMA. Notably, a concomitant downregulation in α2a-AR and α1-AR was detected in mesedin-treated HSCs. These data may reflect that α2a-AR and α1-AR are at least partially involved in the profibrogenic action of NE, which is produced and released by HSCs [2]. HSCs have been previously shown to possess α1-AR, β1-AR, and β2-AR [2]. In addition, during liver fibrosis, only α1-AR and β-AR have been shown to be altered by fibrotic injury in vivo, whereas the involvement of α2-AR remained unexplored. α1-AR is decreased in the liver of patients with liver cirrhosis and portal hypertension [24], while β3-AR is markedly upregulated in a CCl_4_ model of fibrosis and patients with cirrhosis [25]. Studies using the antagonists of α1-AR and β-AR have hinted at the protective features of the α1 and β2 blockade, which in turn has unveiled the role of these receptors in the progression of liver fibrosis [26]. Mesedin appears not only to block α2-AR specifically, but also to reduce the de novo production of α2-AR. Thus, mesedin may exert its antifibrogenic effects via at least two different actions that may, each alone or together, contribute to HSC deactivation: i) via the decrease of α2-AR expression and ii) via the direct blockade of α2-AR.

The α2-AR blockade (in other nonparenchymal cells) has been reported to influence the generation and release of profibrogenic factors. The direct activation of α2-AR by NE has led to a dramatic increase in TNFα in Kupffer cells, which was reversed by the α2-AR antagonist yohimbine [7]. Our data showing a decrease in the key fibrotic marker TGF-β due to mesedin in HSCs hint at the profibrogenic features of α2-AR in HSCs. Besides decreased TGF-β, the deactivation of HSCs by mesedin was confirmed by α-SMA downregulation, which was shown at the RNA and protein level. To date, the modulation of TGF-β by NE in different cell types has been primarily ascribed to α1-AR, as shown in primary rat hepatocyte cultures [27] or in the ventricular myocardium of female and male Sprague–Dawley rats, where the NE-induced expression of the mRNA of all TGF isoforms and type I and type III collagen was reduced by the α1-adrenoceptor blockade [28].

PDGF has been shown to desensitize α1-AR through phosphorylation [29]. Mesedin did not alter the production of PDGF in the HSCs; however, in the hLSECs it appeared to decrease the intensity of α1-AR expression. In the context of previous findings reporting a decrease in the α1-AR density in rat lung membranes in response to the NO donor compound *S*-nitrosoglutathione [30], our data hint at a link between the eNOS and α1-AR expression level in endothelial cells. Future studies employing the inhibitors of NOS may confirm the crosstalk between α2- and α1-AR via NO.

The notion that NE substantially contributes to the progression of liver fibrosis is supported by reports showing elevated levels of NE during nonalcoholic fatty liver disease (NAFLD) or after sepsis or hemorrhagic shock [13,31]. In addition, exogenous NE stimulates proliferation of HSCs, leading to hepatocellular dysfunction [13]. In NAFLD, the expression of α1-, β1-, β2-, and β3-AR is dependent on the stage of fibrosis/cirrhosis: while in an early stage of fibrosis, these receptors are decreased, in progressive fibrosis/cirrhosis, they are markedly upregulated [13]. Moreover, β1-AR has been suggested as being involved in the development of nonalcoholic steatohepatitis [32], while α2-AR remains uninvestigated.

One of the most important hallmarks of liver fibrosis/cirrhosis is the decrease in sinusoidal endothelial cell permeability [22]. In mesedin-treated hLSECs, the permeability marker eNOS increases concomitantly with the downregulation of TGFβ. Endothelial dysfunction is characterized by the decreased bioavailability of NO in cirrhotic livers, suggesting that NO as a possible target for the treatment of liver fibrosis [33,34]. Notably, the inhibition of collagen I, α-SMA, and fibrogenic genes in HSCs has previously been observed in primary rat liver and human activated HSCs treated with nanoparticles containing NO donor molecules releasing NO [35]. Thus, the restoration of eNOS activity in mesedin-treated cells can be considered to be a valuable strategy for NO induction in hLSECs. In contrast to other nonparenchymal cells of the liver, the expression of α2-AR in hLSECs remains unexplored. α2-AR expression has been reported in HSCs, Kupffer cells, hepatocytes [4,5,6,7], and endothelial cells of extrahepatic origin [1,36]. The α2-AR-mediated modulation of NO generation by endothelial cells has been recently proven by Chen et al., showing a decrease in renal NO/inducible NOS (iNOS) in response to the α2-AR agonist dexmedetomidine [37]. However, other reports have suggested an NO-enhancing role for α2-AR agonists, proposing different mechanisms for the crosstalk between α- and β-AR and endothelial NOS (for a review, see Reference [38]). Since the relaxation of endothelium induced by β-adrenergic agonists is prevented by inhibitors of NOS [38], one possible pathway for a mesedin-induced increase in eNOS, besides the direct deactivation of α2-AR, can be via a NE effect on β-AR. In summary, previous findings along with our data demonstrating an increase in eNOS in hLSECs due to mesedin, hint at a role for α2-AR in the regulation of NO production through either iNOS or eNOS, depending on the type of targeted cells.

Hypoxia has been shown to affect approximately one-third of patients with chronic liver disease and is considered to be a prognosis-worsening factor [39]. In mice, the hypoxia-inducible factor 1α (HIF-1α) generated upon oxygen deprivation has been demonstrated to be a crucial factor in collagen cross-linking, contributing to liver fibrosis in NAFLD [40]. In neural cells, an α2-AR blockade due to mesedin has led to the improved survival of astroglia, neurons, and neuronal progenitors, mediated partially by the upregulation of interleukin-10 (IL-10) [11]. Extrapolating this result to the progression of liver injury, it can be assumed that mesedin may exert its antifibrotic properties through IL-10, which is known to possess strong protective features in hepatocytes and to induce the senescence of activated HSCs [41,42]. By acknowledging the antihypoxic properties of mesedin in the central nervous system in vitro and in vivo [9,10,11], future investigations of mesedin’s effects on parenchymal and nonparenchymal liver cells exposed to hypoxia can provide additional mechanistic insight into the function of hepatic α2-AR and the feasibility of its blockade in the treatment of chronic liver injury.

## Figures and Tables

**Table 1:** TaqMan gene expression assays used for the mRNA analysis.

Gene Alias	Gene Symbol	RefSeq	Assay ID
α-SMA	*Acta2*	NM_007392.3	Mm01204962_gH
α2a-AR	*Adra2a*	NM_007417.4	Mm00845383_s1
α2b-AR	*Adra2b*	NM_009633.3	Mm00477390_s1
α2c-AR	*Adra2c*	NM_007418.3	Mm00431686_s1
GAPDH	*Gapdh*	NM_008084.3; NM_001289726.1	Mm99999915_g1

**Table 2:** Antibodies used for Western blot and immunofluorescence analyses.

Antibody	Catalog Number	Supplier
α-SMA (Western) (rabbit monoclonal)	ab124964	abcam, Cambridge, UK
α-SMA (IHC) (mouse monoclonal)	61001	Progen, Heidelberg, Germany
TGF-β (rabbit polyclonal)	ab92486	abcam
α2a-AR (rabbit polyclonal)	SAB4500548	Sigma-Aldrich, Taufkirchen, Germany
α1-AR (rabbit polyclonal)	ab3462	abcam
eNOS (rabbit monoclonal)	#32027	Cell Signaling Technology, Frankfurt am Main, Germany
PDGF-AA (rabbit polyclonal)	ab135881	abcam
Goat antimouse IgG Fluorescein isothiocyanate (FITC)-conjugated (polyclonal)	115-095-003	Jackson ImmunoResearch, Ely, UK
Goat antirabbit IgG Cy3-conjugated (polyclonal)	111-165-144	Jackson ImmunoResearch
Goat antirabbit Cy3-conjugated (monoclonal)	28901106V	GE Healthcare UK Ltd., Amersham, Little Chalfont, UK
Goat antimouse Cy5-conjugated (monoclonal)	PA45009V	GE Healthcare UK Ltd.

## References

[1] Henriksen J.H., Møller S., Ring-Larsen H., Christensen N.J. (1998). The sympathetic nervous system in liver disease. J. Hepatol..

[2] Oben J.A., Roskams T., Yang S., Lin H., Sinelli N., Torbenson M., Smedh U., Moran T.H., Li Z., Huang J. (2004). Hepatic fibrogenesis requires sympathetic neurotransmitters. Gut.

[3] Oben J.A., Roskams T., Yang S., Lin H., Sinelli N., Li Z., Torbenson M., Thomas S.A., Diehl A.M. (2003). Norepinephrine induces hepatic fibrogenesis in leptin deficient ob/ob mice. Biochem. Biophys. Res. Commun..

[4] Miksa M., Das P., Zhou M., Wu R., Dong W., Ji Y., Goyert S.M., Ravikumar T.S., Wang P. (2009). Pivotal role of the α2A-adrenoceptor in producing inflammation and organ injury in a rat model of sepsis. PLoS ONE.

[5] Hoffman B.B., Dukes D.F., Lefkowitz R.J. (1981). Alpha-adrenergic receptors in liver membranes: Delineation with subtype selective radioligands. Life Sci..

[6] Bylund D.B. (1992). Subtypes of alpha-1 and alpha-2 adrenergic receptors. Eur. Neuropsychopharmacol..

[7] Zhou M., Yang S., Koo D.J., Ornan D.A., Chaudry I.H., Wang P. (2001). The role of Kupffer cell α2-adrenoceptors in norepinephrine-induced TNF-α production. Biochim. Biophys. Acta-Mol. Basis Dis..

[8] Xuanfei L., Hao C., Zhujun Y., Yanming L., Jianping G. (2017). Imidazoline I2 receptor inhibitor idazoxan regulates the progression of hepatic fibrosis via Akt-Nrf2-Smad2/3 signaling pathway. Oncotarget.

[9] Tananyan A., Balasanyan M.P. (2014). 4.c.008 Prevention of focal ischemia induced memory deficit and anxiety by mesedin. Eur. Neuropsychopharmacol..

[10] Tananyan A.G., Balasanyan M.G., Baykov A.V., Hovsepyan L.M., Ghazaryan G.S. (2019). The effect of mesedin on the content of oxidative stress biomarkers in the brain tissue in ischemia. Neurochem. J..

[11] Melkonyan M.M., Hunanyan L., Lourhmati A., Layer N., Beer-Hammer S., Yenkoyan K., Schwab M., Danielyan L. (2018). Neuroprotective, neurogenic, and amyloid beta reducing effect of a novel alpha 2-Adrenoblocker, mesedin, on astroglia and neuronal progenitors upon hypoxia and glutamate exposure. Int. J. Mol. Sci..

[12] Lin J.C., Peng Y.J., Wang S.Y., Lai M.J., Young T.H., Salter D.M., Lee H.S. (2016). Sympathetic nervous system control of carbon tetrachloride-induced oxidative stress in liver through α-adrenergic signaling. Oxid. Med. Cell. Longev..

[13] Sigala B., McKee C., Soeda J., Pazienza V., Morgan M., Lin C.I., Selden C., Vander Borght S., Mazzoccoli G., Roskams T. (2013). Sympathetic nervous system catecholamines and neuropeptide Y neurotransmitters are upregulated in human NAFLD and modulate the fibrogenic function of hepatic stellate cells. PLoS ONE.

[14] Sha J., Zhang H., Zhao Y., Feng X., Hu X., Wang C., Song M., Fan H. (2019). Dexmedetomidine attenuates lipopolysaccharide-induced liver oxidative stress and cell apoptosis in rats by increasing GSK-3β/MKP-1/Nrf2 pathway activity via the α2 adrenergic receptor. Toxicol. Appl. Pharmacol..

[15] Vartanyan S.O., Avakyan A.S., Sargsyan A.B., Arutyunyan S.A., Noravyan O.S., Tsatinyan A.S. (2016). Synthesis and biologic properties of new thiazolylbenzodioxane derivatives. Russ. J. Org. Chem..

[16] Garcia-Tsao G. (2016). Beta blockers in cirrhosis: The window re-opens. J. Hepatol..

[17] Borkham-Kamphorst E., van de Leur E., Zimmermann H.W., Karlmark K.R., Tihaa L., Haas U., Tacke F., Berger T., Mak T.W., Weiskirchen R. (2013). Protective effects of lipocalin-2 (LCN2) in acute liver injury suggest a novel function in liver homeostasis. Biochim. Biophys. Acta-Mol. Basis Dis..

[18] Tag C., Weiskirchen S., Hittatiya K., Tacke F., Tolba R., Weiskirchen R. (2015). Induction of experimental obstructive cholestasis in mice. Lab. Anim..

[19] Livak K.J., Schmittgen T.D. (2001). Analysis of relative gene expression data using real-time quantitative PCR and the 2-ΔΔCT method. Methods.

[20] Proell V., Mikula M., Fuchs E., Mikulits W. (2005). The plasticity of p19ARF null hepatic stellate cells and the dynamics of activation. Biochim. Biophys. Acta-Mol. Cell Res..

[21] De Salamanca A.E., Siemasko K.F., Diebold Y., Calonge M., Gao J., Juárez-Campo M., Stern M.E. (2005). Expression of muscarinic and adrenergic receptors in normal human conjunctival epithelium. Investig. Ophthalmol. Vis. Sci..

[22] Deleve L.D. (2015). Liver sinusoidal endothelial cells in hepatic fibrosis. Hepatology.

[23] Yoshida M., Nishikawa Y., Omori Y., Yoshioka T., Tokairin T., McCourt P., Enomoto K. (2007). Involvement of signaling of VEGF and TGF-β in differentiation of sinusoidal endothelial cells during culture of fetal rat liver cells. Cell Tissue Res..

[24] Zhu J., Chen L., Leng X., Du R. (2000). Expression of alpha1 adrenoceptor subtypes mRNA in hepatic tissues of cirrhotic patients with portal hypertension. Zhonghua Wai Ke Za Zhi.

[25] Trebicka J., Hennenberg M., Pröbsting A.S., Laleman W., Klein S., Granzow M., Nevens F., Zaagsma J., Heller J., Sauerbruch T. (2009). Role of β3-adrenoceptors for intrahepatic resistance and portal hypertension in liver cirrhosis. Hepatology.

[26] Muñoz-Ortega M.H., Llamas-Ramírez R.W., Romero-Delgadillo N.I., Elías-Flores T.G., De Jesus Tavares-Rodríguez E., Del Rosario Campos-Esparza M., Cervantes-García D., Muñoz-Fernández L., Gerardo-Rodríguez M., Ventura-Juárez J. (2016). Doxazosin treatment attenuates carbon tetrachloride-induced liver fibrosis in hamsters through a decrease in transforming growth factor β secretion. Gut Liver.

[27] Fabregat I., Moreno-Càceres J., Sánchez A., Dooley S., Dewidar B., Giannelli G., ten Dijke P. (2016). TGF-β signalling and liver disease. FEBS J..

[28] Briest W., Homagk L., Raßler B., Ziegelhoffer-Mihalovicova B., Meier H., Tannapfel A., Leiblein S., Saalbach A., Deten A., Zimmer H.G. (2004). Norepinephrine-induced changes in cardiac transforming growth factor-β isoform expression pattern of female and male rats. Hypertension.

[29] Medina L.D.C., Vazquez-Prado J., Garcia-SHIRSCH-ERNSTainz J.A. (2000). Cross-talk between receptors with intrinsic tyrosine kinase activity and alpha1b-adrenoceptors [In Process Citation] 154 154. Biochem. J..

[30] Nozik-Grayck E., Whalen E.J., Stamler J.S., McMahon T.J., Chitano P., Piantadosi C.A. (2006). S-nitrosoglutathione inhibits α1-adrenergic receptor-mediated vasoconstriction and ligand binding in pulmonary artery. Am. J. Physiol.-Lung Cell. Mol. Physiol..

[31] Wang P., Tait S.M., Chaudry I.H. (2000). Sustained elevation of norepinephrine depresses hepatocellular function. Biochim. Biophys. Acta-Mol. Basis Dis..

[32] Ueta C.B., Fernandes G.W., Capelo L.P., Fonseca T.L., Maculan F.D.A., Gouveia C.H.A., Brum P.C., Christoffolete M.A., Aoki M.S., Lancellotti C.L. (2012). β1 Adrenergic receptor is key to cold-and diet-induced thermogenesis in mice. J. Endocrinol..

[33] Vairappan B. (2015). Endothelial dysfunction in cirrhosis: Role of inflammation and oxidative stress. World J. Hepatol..

[34] Bosch J., Abraldes J.G., Fernández M., García-Pagán J.C. (2010). Hepatic endothelial dysfunction and abnormal angiogenesis: New targets in the treatment of portal hypertension. J. Hepatol..

[35] Duong H.T.T., Dong Z., Su L., Boyer C., George J., Davis T.P., Wang J. (2015). The use of nanoparticles to deliver nitric oxide to hepatic stellate cells for treating liver fibrosis and portal hypertension. Small.

[36] Angus J.A., Cocks T.M., Satoh K. (1986). The α adrenoceptors on endothelial cells. Fed. Proc..

[37] Chen Y., Luan L., Wang C., Song M., Zhao Y., Yao Y., Yang H., Ma B., Fan H. (2019). Dexmedetomidine protects against lipopolysaccharide-induced early acute kidney injury by inhibiting the iNOS/NO signaling pathway in rats. Nitric Oxide-Biol. Chem..

[38] Vanhoutte P.M. (2001). Endothelial adrenoceptors. J. Cardiovasc. Pharmacol..

[39] Rao M.Y., Raghu J., Deshmukh S., Amaravathi K.S., Sudhir U. (2008). Arterial hypoxemia in patients with cirrhosis of liver. J. Assoc. Physicians India.

[40] Mesarwi O.A., Shin M.K., Bevans-Fonti S., Schlesinger C., Shaw J., Polotsky V.Y. (2016). Hepatocyte hypoxia inducible factor-1 mediates the development of liver fibrosis in a mouse model of nonalcoholic fatty liver disease. PLoS ONE.

[41] Abd-Elgawad H., Abu-Elsaad N., El-Karef A., Ibrahim T. (2016). Piceatannol increases the expression of hepatocyte growth factor and IL-10 thereby protecting hepatocytes in thioacetamide-induced liver fibrosis. Can. J. Physiol. Pharmacol..

[42] Huang Y.-H., Chen M.-H., Guo Q.-L., Chen Z.-X., Chen Q.-D., Wang X.-Z. (2020). Interleukin-10 induces senescence of activated hepatic stellate cells via STAT3-p53 pathway to attenuate liver fibrosis. Cell. Signal..

